# Characterization of functional subgroups among genetically identified cholinergic neurons in the pedunculopontine nucleus

**DOI:** 10.1007/s00018-019-03025-4

**Published:** 2019-02-08

**Authors:** B. Baksa, A. Kovács, T. Bayasgalan, P. Szentesi, Á. Kőszeghy, P. Szücs, Balázs Pál

**Affiliations:** 1 Department of Physiology, University of Debrecen, Faculty of Medicine, Nagyerdei krt 98, Debrecen, 4012 Hungary; 2 Department of Anatomy, Histology and Embriology, University of Debrecen, Faculty of Medicine, Debrecen, Hungary; 3 Present Address: Division of Cognitive Neurobiology, Center for Brain Research, Medical University of Vienna, Vienna, Austria

**Keywords:** Pedunculopontine nucleus, Cholinergic neuron, Spike delay, A-current, Rostrocaudal gradient, High threshold oscillation

## Abstract

The pedunculopontine nucleus (PPN) is a part of the reticular activating system which is composed of cholinergic, glutamatergic and GABAergic neurons. Early electrophysiological studies characterized and grouped PPN neurons based on certain functional properties (i.e., the presence or absence of the A-current, spike latency, and low threshold spikes). Although other electrophysiological characteristics of these neurons were also described (as high threshold membrane potential oscillations, great differences in spontaneous firing rate and the presence or absence of the M-current), systematic assessment of these properties and correlation of them with morphological markers are still missing. In this work, we conducted electrophysiological experiments on brain slices of genetically identified cholinergic neurons in the PPN. Electrophysiological properties were compared with rostrocaudal location of the neuronal soma and selected morphometric features obtained with post hoc reconstruction. We found that functional subgroups had different proportions in the rostral and caudal subregions of the nucleus. Neurons with A-current can be divided to early-firing and late-firing neurons, where the latter type was found exclusively in the caudal subregion. Similar to this, different parameters of high threshold membrane potential oscillations also showed characteristic rostrocaudal distribution. Furthermore, based on our data, we propose that high threshold oscillations rather emerge from neuronal somata and not from the proximal dendrites. In summary, we demonstrated the existence and spatial distribution of functional subgroups of genetically identified PPN cholinergic neurons, which are in accordance with differences found in projection and in vivo functional findings of the subregions. Being aware of functional differences of PPN subregions will help the design and analysis of experiments using genetically encoded opto- and chemogenetic markers for in vivo experiments.

**Electronic supplementary material:**

The online version of this article (10.1007/s00018-019-03025-4) contains supplementary material, which is available to authorized users.

## Introduction

The pedunculopontine nucleus (PPN), together with the laterodorsal tegmental nucleus form the mesopontine cholinergic areas (also known as Ch5 and 6; [1]). The classical view of its function is, as a part of the reticular activating system (RAS), that it is a significant modulator of the sleep–wakefulness states, as well as of the switch between non-REM and REM sleep, and that it participates in sensory gating and locomotion regulation. Modulation of sleep–wakefulness cycles was thought to be critically dependent on cholinergic neurons. However, recent studies using novel transgenic techniques and selective stimulation of neurochemically distinct neuronal populations led to sometimes contradictory results on the significance of cholinergic neurons on initiation and maintenance of REM sleep. Based on the recent data, cholinergic actions of the PPN can be rather understood as an initiator of behavioral switch [2–6].

Involvement of the PPN in different neurodegenerative, neuropsychiatric diseases such as dementia with Lewy bodies, Parkinson’s disease (PD) or schizophrenia is well known [5, 7–10]. Furthermore, the PPN is also a target for deep brain stimulation (DBS) in PD with sometimes contradictory outcomes [6, 11].

Besides hosting a large proportion of cholinergic neurons, there are also intermingled populations of GABAergic and glutamatergic neurons in the nucleus. Neurochemically distinct populations exist in different proportions in subterritories of the nucleus: the rostral pars compacta contains less cholinergic and a greater number of GABAergic neurons, whereas the caudal pars compacta is dominated by densely packed cholinergic somata together with higher density of glutamatergic neurons, with a minimal overlap between the latter two populations [12–15].

Besides neurochemical groups based on neurotransmitter content [12, 14, 15], differences in distribution of calcium-binding proteins (rostrocaudal gradient of calbindin and calretinin; [16]) or the presence of certain neuropeptides (e.g., galanin, [5, 17]) were also suggested to define subgroups of PPN neurons. NADPH diaphorase, neuronal nitric oxide synthetase (bNOS) and choline acetyltransferase were investigated as putative cholinergic PPN neuron markers [18–20], from which bNOS was recently proven to be a highly selective marker for cholinergic neurons [21].

Functional groups were also described in in vitro experiments, where the sorting criteria were the presence or absence of low threshold calcium spikes and transient outward potassium current (known as A-current) [19, 20, 22]. Type I neurons possess low threshold calcium spikes (LTS), type II neurons had A-current and low threshold spike (A-type), whereas type III neurons possessed none of them [22] or both of them (A+LTS) in other classifications [19, 20].

High threshold oscillations (HTOs) are another important characteristics of certain PPN neurons. The phenomenon was originally demonstrated by Takakusaki et al. [23], where low amplitude–high frequency and high amplitude–low frequency activities were identified on type II cholinergic neurons. Oscillatory activity seemed to correlate with spike duration and spontaneous firing frequency. Existence of HTOs on PPN cholinergic neurons was confirmed by more recent papers [24, 25].

It was also recently shown that the existence of M-current is a characteristic for cholinergic neurons, and other neurochemical subpopulations do not possess this feature [25].

PPN cholinergic neurons in vivo were shown to discharge with higher rates during wakefulness and REM sleep than during slow wave activity [26] and a greater proportion of cholinergic neurons (the ‘slow firing’ neurons) display an activity temporally correlated with nested gamma oscillations of cortical slow wave activity, whereas a smaller population (the ‘fast firing’ neurons) lacks this correlation [27].

Along its rostrocaudal axis, the PPN is not homogenous. Several morphological and functional differences were described along this axis, using different methods ranging from histological analysis, through in vivo recording to analyzing clinical findings. Neuronal density, neurochemical markers or projections, as well as association of caudal and rostral PPN to different functions and tasks show marked differences [2, 6, 13, 16, 17, 28–31].

Despite its potential importance in understanding results of in vivo stimulation of different PPN neuronal populations or in the design and assessment of DBS in PD, topographical differences in PPN cholinergic neuronal membrane properties are poorly understood. In the present work, we tried to correlate rostrocaudal location of PPN cholinergic neurons with their functional properties, using a mouse model expressing a genetically encoded indicator.

We showed that the grouping criteria suggested by earlier studies can be used to separate PPN cholinergic neurons in our transgenic model. In addition, rostrocaudal differences in low threshold spikes, A-current kinetics and HTOs were also seen. These topographical differences might explain the inhomogeneity in PD pathophysiology and in results of DBS.

## Materials and methods

### Solutions, chemicals

Artificial cerebrospinal fluid (aCSF) used for slice electrophysiology experiments had the following composition (in mM): NaCl, 120; KCl, 2.5; NaHCO_3_, 26; glucose, 10; NaH_2_PO_4_, 1.25; myo-inositol, 3; ascorbic acid, 0.5; sodium-pyruvate, 2; CaCl2, 2; MgCl_2_, 1; pH 7.2. Acute slices were prepared in low Na^+^ aCSF, where 95 mM NaCl was replaced by glycerol (60 mM) and sucrose (130 mM). All chemicals were purchased from Sigma (St. Louis, MO, USA), unless stated otherwise.

### Animals, preparation

Animal experiments were conducted in accordance with the appropriate national and international (EU Directive 2010/63/EU for animal experiments) institutional guidelines and laws on the care of research animals. Experimental protocols were approved by the Committee of Animal Research of the University of Debrecen (5/2015/DEMÁB; 8/2015/DEMÁB). 12- to 22-day-old mice expressing tdTomato fluorescent protein in a choline acetyltransferase (ChAT) dependent way (*n* = 49) from both sexes were employed. To get these mice, homozygous floxed stop tdTomato (B6;129S6-Gt(ROSA)26Sor^tm9(CAG−tdTomato)Hze/^J; Jax mice Accession Number: 007905) and ChAT-cre (B6;129S6-Chat^tm2(cre)Lowl/^J; Jax Number: 006410) strains purchased from Jackson Laboratories (Bar Harbor, ME, USA) were crossed in our animal facility. Mice expressing tdTomato in a type 2 vesicular glutamate transporter (Vglut2) dependent way were obtained by crossing the tdTomato line with homozygous Vglut2-cre (*Slc17a6*^*tm2(cre)Lowl*^ (also called Vglut2-ires-Cre); Jax Number: 028863) mice. Midbrain slices (coronal plane, 200 μm thickness) were prepared in ice-cold (cca. 0 to − 2 °C) low Na^+^ aCSF with a Microm HM 650 V vibratome (Microm International GmbH, Walldorf, Germany). The slices were kept in normal aCSF for 1 h on 37 °C prior to starting the experiment.

### Electrophysiology

The resistance of the patch pipettes was 6–8 MΩ, and the composition of the internal solution was the following (in mM): K-gluconate, 120; NaCl, 5; 4-(2-hydroxyethyl)-1-piperazineethanesulfonic acid (HEPES), 10; Na_2_-phosphocreatinine, 10; EGTA, 2; CaCl_2_, 0.1; Mg-ATP, 5; Na_3_-GTP, 0.3; biocytin, 8; pH 7.3. Whole-cell patch-clamp experiments were conducted at room temperature (24–26 °C) on neuronal somata with an Axopatch 200A amplifier (Molecular Devices, Union City, CA, USA). Data acquisition was achieved with Clampex 10.0 software (Molecular Devices, Union City, CA, USA), while data analysis was performed by Clampfit 10.0 (Molecular Devices) software. Only stable recordings with minimal leak currents were considered and only recordings with series resistance below 30 MΩ, with less than 10% change were included.

Both voltage- and current-clamp configurations were used. In certain experiments, 1 μM tetrodotoxin (TTX; Alomone Laboratories, Jerusalem, Israel) or 50 μM CdCl_2_ was used to eliminate action potential generation and calcium channel-mediated actions, respectively.

Protocols detailed below were used to assess functional parameters of PPN cholinergic neurons (Suppl. Fig 1.). In current-clamp configuration, 1-s-long square current pulses were used between − 30 pA and + 120 pA with 10 pA increment. The resting membrane potential was set to − 60 or − 80 mV, depending on the type of experiment. Input resistance was calculated from the difference between the average voltages of 10 ms long periods prior to and at the end of the trace corresponding to the − 30 pA steps (Suppl. Fig 1A). At different depolarizing pulses, action potential firing frequency of the entire trace during the square pulse was considered. The maximal firing frequency of the neuron was assessed from the shortest interspike interval recorded during the depolarizing steps (in most cases, between the first two action potentials of the + 120 pA square pulse). The spike latency of recordings at membrane potentials of − 60 and − 80 mV was calculated as the time difference between the beginning of the current step and the peak potential of the first action potential at the sweep recorded with 100 pA depolarizing current injection. Adaptation index (AI) was calculated using the following formula: AI = 1 − (*F*_last_/*F*_initial_), where *F*_last_ is the average frequency of the last two action potentials and *F*_initial_ is the average frequency of the first three action potentials (Suppl. Fig 1B; [32]).

**Fig. 1:**
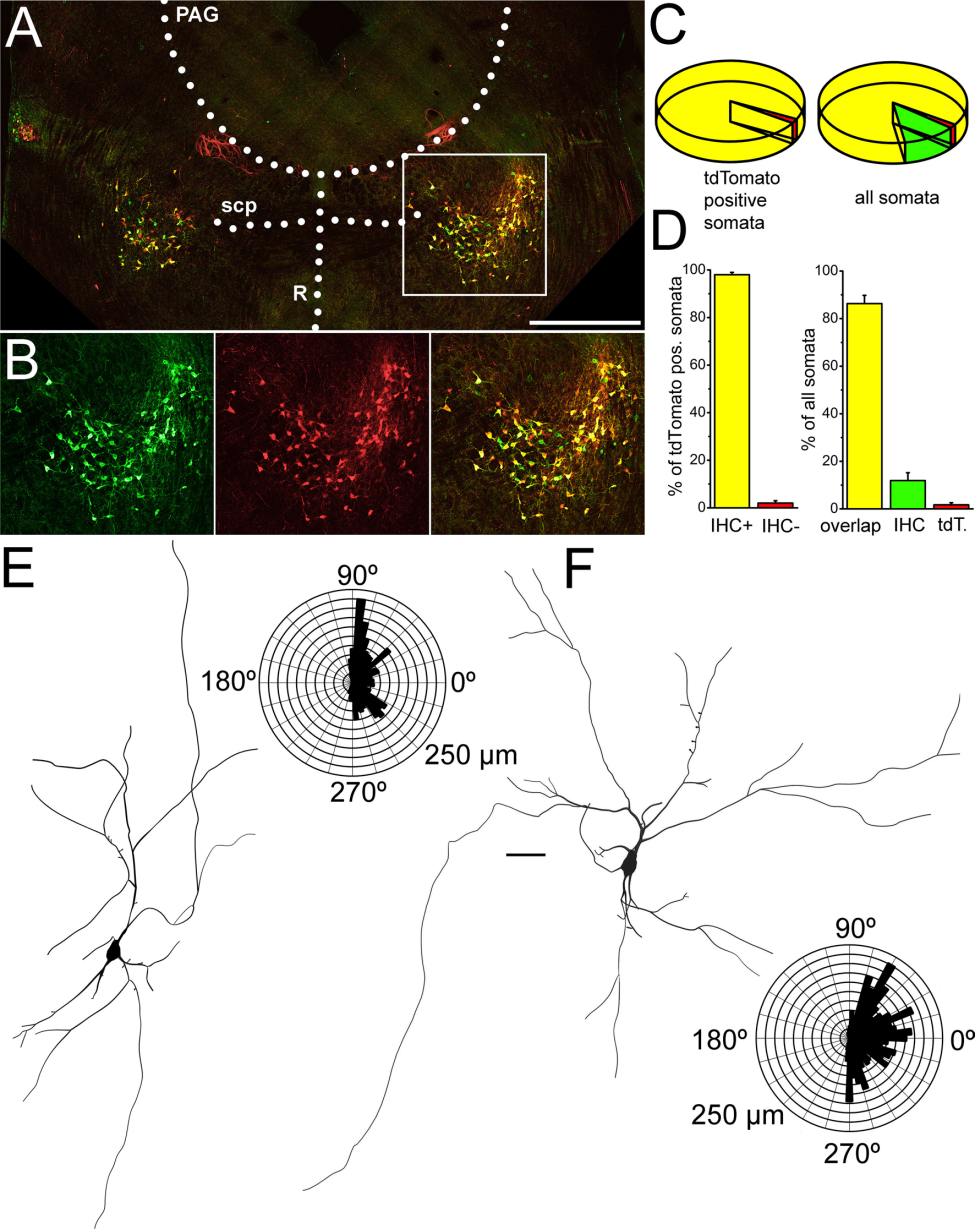
Morphological evaluation of samples used for functional experiments. **a**–**d** Choline acetyltransferase immunohistochemistry almost fully overlaps with tdTomato expression driven by choline acetyltransferase promoter. **a** Overview of a coronal section from a ChAT-tdTomato animal after performing ChAT immunohistochemical labeling (red: tdTomato, green: ChAT immunohistochemistry, PAG: periaqueductal grey matter, scp: superior cerebellar peduncle, R: dorsal and median raphe nuclei). Scale bar: 1 mm. **b** Magnified images of the area indicated by the white square on **a**. **c** Pie diagram of the distribution of labeled somata (left, yellow: overlapping labeling, green: only immunohistochemical labeling, red: only tdTomato expression). Pie diagram of tdTomato positive somata (yellow: ChAT positivity confirmed by immunohistochemistry, red: tdTomato positive somata with no ChAT immunopositivity). **d** Statistical analysis of the proportions of different somata (obtained from three animals) with the same arrangement as on **c**. **e**, **f** Morphological characteristics of ChAT-tdTomato positive neurons labeled during slice electrophysiology experiments. **e** Soma and dendritic tree of a cholinergic neuron with bipolar dendritic tree. **f** Soma and dendritic tree of a cholinergic neuron with multipolar dendritic tree. Scale bar: 50 µm

Low threshold spikes (LTS) were defined as the maximal voltage differences between the peaks of transient depolarizations and the average steady state voltages at the last 100 ms of the depolarizing step; measured in the presence of TTX. Rebound spikes were assessed as maximal voltage differences of the transient depolarization following − 30 mV hyperpolarizing voltage step and the resting membrane potential measured as the average voltage of a 100-ms-long trace preceding the voltage step.

Transient outward potassium currents (known as A-current, [33, 34]) were recorded in voltage clamp configuration, with a 1-s-long depolarizing square pulse of + 20 mV preceded by 200-ms-long prepulses of − 120 and 0 mV; from a holding potential of − 60 mV. The difference between the two current traces at the depolarizing pulse was considered as A-current. The declining phase of the transient outward current was fit with a single exponential function (Suppl. Fig 1C).

HTOs were recorded in current-clamp mode from − 60 mV with a 2-s-long depolarizing ramp protocol from 0 pA to 800 pA [24, 35], in the presence of TTX to eliminate action potential firing. Power spectra of the whole traces during the depolarizing steps were assessed and power maximum and frequency belonging to power maximum were determined (Suppl. Fig 1D–F).

In certain cases, simultaneous recordings were performed on neuronal somata and proximal dendrites (29 ± 4 µm away from the soma) using patch pipettes with 6–8 MΩ resistance.

Visualization of the genetically encoded fluorescent marker (tdTomato) was achieved using a fluorescent imaging system (Till Photonics GmbH, Gräfeling, Germany) containing a xenon bulb-based Polychrome V light source, a CCD camera (SensiCam, PCO AG, Kelheim, Germany), an imaging control unit (ICU), and the Till Vision software (version 4.0.1.3).

### Morphological analysis and reconstruction of the recorded neurons

Patched neurons were labeled with biocytin and samples were fixed (4% paraformaldehyde in 0.1 M phosphate buffer; pH 7.4; 4 °C) for morphological identification of the neurons. Tris-buffered saline (in mM, Tris base, 8; Trisma HCl, 42; NaCl, 150; pH 7.4) supplemented with 0.1% Triton X-100 and 10% bovine serum (60 min) was used for permeabilization. Incubation was performed in phosphate buffer containing streptavidin-conjugated Alexa488 (1:300; Molecular Probes Inc., Eugene, OR, USA) for 90 min.

After the labeling procedure, the visualization of the neurons was achieved using confocal microscope (Zeiss LSM 510; Carl Zeiss AG); tile scan images were taken with 40x objective and with 1 μm optical slices. The reconstruction of neurons was performed by NeuroLucida software (MBF Bioscience, Williston, VT, USA). For preparation of the topographical maps of functional properties, the three-dimensional location of the individual neurons (including the rostrocaudal location of the slice containing the labeled neuron) was determined on the basis of the Paxinos atlas [36]. A three-dimensional block reconstruction using the contours of the brainstem and the cerebral aqueduct was made using the adequate contours and parameters of the atlas, and positions of cell bodies were indicated with spheres. Color codes of these spheres represent various functional parameters; described below in the adequate chapters of ‘Results’.

### Choline acetyltransferase immunohistochemistry

In three cases, choline acetyltransferase immunohistochemistry was performed on sections from adult (3- to 6-month-old) transcardially perfused ChAT-tdTomato mice. Brain samples were post-fixed in 4% paraformaldehyde for 24 h and 80-μm-thick coronal slices were prepared. After three times 10-min washing with Tris-buffered saline (TBS), the slices were permeabilized with 10% Triton-X100 and 0.5% bovine serum for 1 h. Anti-choline acetyltransferase primary antibodies from rabbit (Millipore, Temecula, CA, USA) were employed in 1:75 for 48 h. Goat anti-rabbit Alexa 488 antibody (Vector Laboratories Inc., Burlingame, CA, USA) was used for another 24 h. After completion of the protocol above, confocal images were taken in a similar way described in Chapter 2.4. Overlaps of tdTomato expression and ChAT immunopositivity were judged manually by counting neuronal somata showing red and green fluorescence.

Slices from Vglut2-tdTomato mice underwent simultaneous biocytin recovery and ChAT immunohistochemistry using the same concentration of chemicals and antibodies as detailed above.

All data represent mean ± SEM. The normal distribution of the datasets was evaluated with D’Agostino and Pearson omnibus normality test. Student’s *t* test was applied for assessing statistical significance for pairwise comparison in case if the datasets had normal distribution, whereas Kolmogorov–Smirnov test was used when datasets lacked normal distribution. For multiple comparisons, Bonferroni’s multiple comparison test was employed. The level of significance was *p *< 0.05. Pearson’s correlation coefficient (*r*) was applied to assess the linear correlation between two independent datasets.

## Results

According to classical studies performed on non-transgenic rats, the PPN neurons were classically grouped to three or four functional categories. The cholinergic and non-cholinergic natures of the neurons were evaluated with bNOS- or ChAT-immunohistochemistry. In the present paper, we aimed to revise the functional grouping of the PPN cholinergic neurons and to test whether the ‘canonical’ functional subgroups are valid for transgenic mouse models. Furthermore, other possibilities for functional grouping were sought and possible correlations between morphological, topographical and functional data were evaluated.

### Identification of cholinergic neurons

First, we aimed to confirm that tdTomato is expressed in genuine cholinergic PPN neurons and that it is not a remnant of a transient ChAT expression during an earlier phase of development. ChAT immunohistochemistry was performed on sections containing the PPN from 3 ChAT-tdTomato mice (4 sections/animal); and 379 somata were evaluated in total. A substantial overlap of tdTomato expression and ChAT immunopositivity was found: 98 ± 1% of all tdTomato-positive somata proved to be ChAT positive. The remaining 2% ± 1% of ChAT immunonegative somata, interestingly, tended to be located at the edges of the PPN.

When not only tdTomato positive somata but all somata positive to any labeling were considered in the territory of the PPN, 86.8 ± 3.5% of the somata showed co-localization of ChAT immunolabeling and TdTomato expression, 11.9 ± 3.3% was only immunopositive to ChAT while 1.76% ± 0.9% showed exclusively tdTomato expression (average of data from individual animals ± SEM; Fig. 1a–d).

Taken together, tdTomato expressed under the promoter of choline acetyltransferase seems to be a reliable marker of cholinergic neurons, with low chance of negative and negligible chance of positive errors. Nevertheless, a negligible number of cholinergic neurons was potentially not considered in our study due to the lack of tdTomato expression. One also cannot exclude that one or two of the total 91 neurons in our study were potentially tdTomato-expressing non-cholinergic neurons. To minimize the number of false positive cholinergic neurons, cells along the edge of the nucleus were avoided.

### Morphometric analysis of cholinergic neurons

During the recordings, neurons were labeled with biocytin and in 14 cases, where reconstruction of most of the somatodendritic domain was possible, underwent morphological analysis. Somatodendritic parameters did not show correlation with rostrocaudal location of the cell bodies. Similarly, somatodendritic parameters did not show any linear correlation with each other; with the exception of evident correlations as the relationship of input resistance and soma diameter or the linear correlations of total dendritic length and number of dendritic nodes and ends (Suppl. Fig 2.). The average longest diameter of somata was 27.04 ± 1.79 µm (ranging from 20.1 to 37.5 µm) and the cell body area was 276.9 ± 27.4 µm^2^ (162–372.5 µm^2^). The average total dendritic length was 1869.42 ± 291 µm (ranging from 945.8 to 3852.1 µm). Neurons had 3–6 primary dendrites (3.8 ± 0.29), with 5–26 nodes (13.3 ± 1.9) and 9–32 ends (16.8 ± 2.13). 28.6% of all neurons possessed a few dendritic spines (2.4 ± 1.25; Fig. 1e, f).

Dendritic orientation was judged on 17 reconstructed neurons. 4 of these neurons had bipolar dendritic tree, as the longer dendrites were oriented to two opposite directions (Fig. 1e), whereas the rest of the reconstructed neurons was multipolar (Fig. 1f). The orientation of the dendrites did not correlate with most functional parameters, with the exception of the presence or absence of low threshold spikes (see below).

Taken together, the majority of somatodendritic parameters of cholinergic neurons seemed to be independent from other morphometric and most of the functional properties of the neurons. Although slice electrophysiological approach with coronal slices resulted somatodendritic parameters comparable with data from in vivo labelings from rat [27], it did not allow thorough analysis of the axonal arborization of these neurons which could possibly reveal important differences between them.

### ‘Classical’ sorting of neurons according to in vitro functional characteristics

**Fig. 2:**
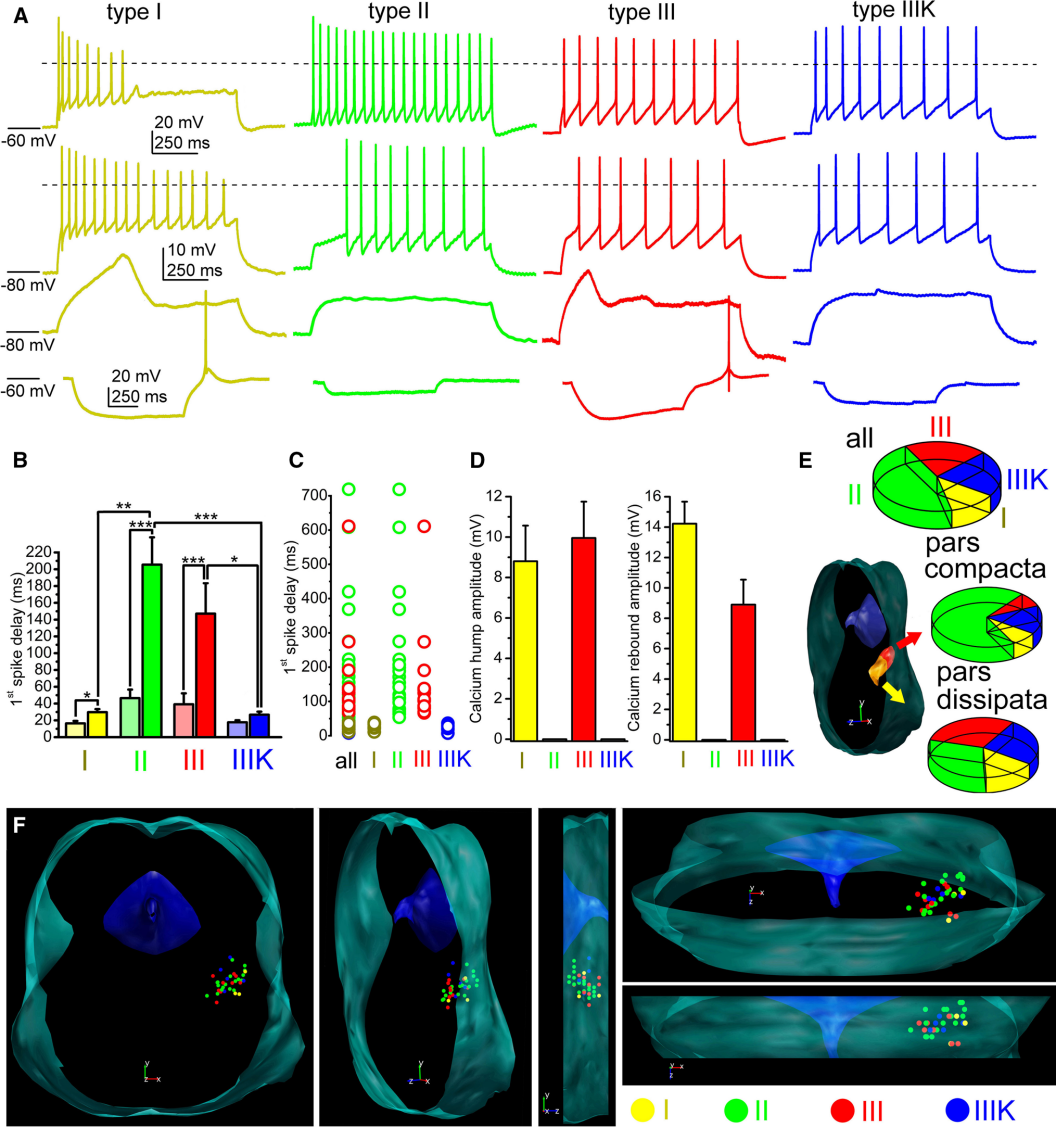
Distribution of functional cell types among cholinergic neurons of the PPN. **a** Voltage traces obtained from different functional types of PPN cholinergic neurons (type I = dark yellow; type II = green; type III = red; type IIIK = blue). Upper row: voltage traces recorded from − 60 mV resting membrane potential with 100 pA depolarizing square current injection. Second row: voltage traces recorded from − 80 mV resting membrane potential with 100 pA depolarizing square current injection. Third row: voltage traces at subthreshold voltages recorded from − 80 mV resting membrane potential with 30 pA depolarizing square current injection. Note the low threshold spikes. Bottom row: voltage traces recorded from − 60 mV resting membrane potential with − 30 pA hyperpolarizing square current injection. Note the rebound spikes and action potentials. Dashed lines indicate 0 mV. **b** Statistical comparison of the delays of first action potentials obtained with 100 pA current injections using the same color codes as on the previous panels. Columns at the left with light colors indicate delays recorded from − 60 mV, whereas columns at the right with darker colors are data obtained from − 80 mV resting membrane potentials (average ± SEM). **c** Individual data of the delay of the first spikes recorded from − 80 mV resting membrane potentials. **d** Statistical summary of the maximal amplitudes of low threshold and rebound spikes, respectively (using the same color code as above). **e** Proportions of functional subtypes in the total population (upper pie diagram), in the pars compacta and dissipata (see reconstruction map; middle and bottom pie diagrams; I = yellow; II = green; III = red; IIIK = blue). **f** Distribution map of functional subtypes in the PPN viewed from different angles (I = yellow; II = green; III = red; IIIK = blue)

We grouped genetically identified cholinergic neurons with functional recordings into four functional sub-groups, according to the ‘canonical’ sorting criteria (*n* = 91) [18, 19, 22, 37, 38]. Neurons with LTS and rebound spikes (caused by calcium conductances) but without A-current and hyperpolarization-induced spike latency were considered as type I neurons. Type II neurons lacked LTS but had A-current and hyperpolarization-induced spike latency. Type III neurons possessed both properties (i.e., delay and LTS as well), whereas type III neurons according to Kang and Kitai ([22]; named as IIIK) lacked both properties (Fig. 2a–d).

In practice, the sorting procedure was the following. The presence or absence of LTS or rebound spikes was evaluated, and the spike latency at 100 pA depolarizing step from -80 mV holding potential was checked. If the latency was less than 50 ms (ranging from 11.3 to 39 ms), but LTSs existed, the neuron was considered as ‘type I’ (*n* = 12; 13.18%). If no LTS but latency greater than 50 ms was found, the neuron was classified as ‘type II’ (ranging from 53.4 to 718.9 ms; *n* = 44; 48.35%). If both phenomena were seen, the neuron was recognized as ‘type III’ (with latencies from 66.8 to 611 ms; *n* = 19; 20.88%), whereas neurons lacking both phenomena were considered as ‘type IIIK’ (with latencies from 7.9 to 36.8 ms; *n* = 16; 17.58%; Fig. 2a–d).

When the spike latencies were compared at − 60 and − 80 mV, a significant increase was seen in all groups. In type I neurons, the delay increased from 16.34 ± 2.66 ms to 29.2 ± 3.44 ms (*p* = 0.02); in type II neurons, from 46.1 ± 10.33 to 199.63 ± 32.05 ms (*p* < 0.001), in type III neurons, from 39.1 ± 13.1 to 147.1 ± 36.3 ms (*p* < 0.001), and in type IIIK neurons, from 17.7 ± 2.4 to 26.7 ± 3.6 ms (statistically not significant). In neurons lacking inactivating transient depolarizing currents, less than twofold increase of the latency was found which is possibly due to passive membrane properties of the given neurons (*n* = 29). In those groups, where there was a potential inactivating transient depolarizing current (and its inactivation was removed by hyperpolarization), a three–fivefold increase in the spike latency was found (*n* = 65). Delays of certain neuronal groups were significantly different (Fig. 2b; I–II, *p* = 0.0017; II–IIIK, *p* = 0.0006; III–IIIK, *p* = 0.045).

In accordance with the original sorting criteria, low threshold and rebound spikes of type I neurons had an amplitude of 9.98 ± 2.1 mV and 14.1 ± 1.3 mV, respectively (*n* = 12). In type III neurons (*n* = 19), low threshold and rebound spikes had amplitude of 9.9 ± 1.8 and 8.9 ± 1.6 mV, respectively. Type II and IIIK neurons—as it is expected from sorting criteria—had no LTS or rebound spike (*n* = 44 and 16, respectively; Fig. 2d).

**Fig. 3:**
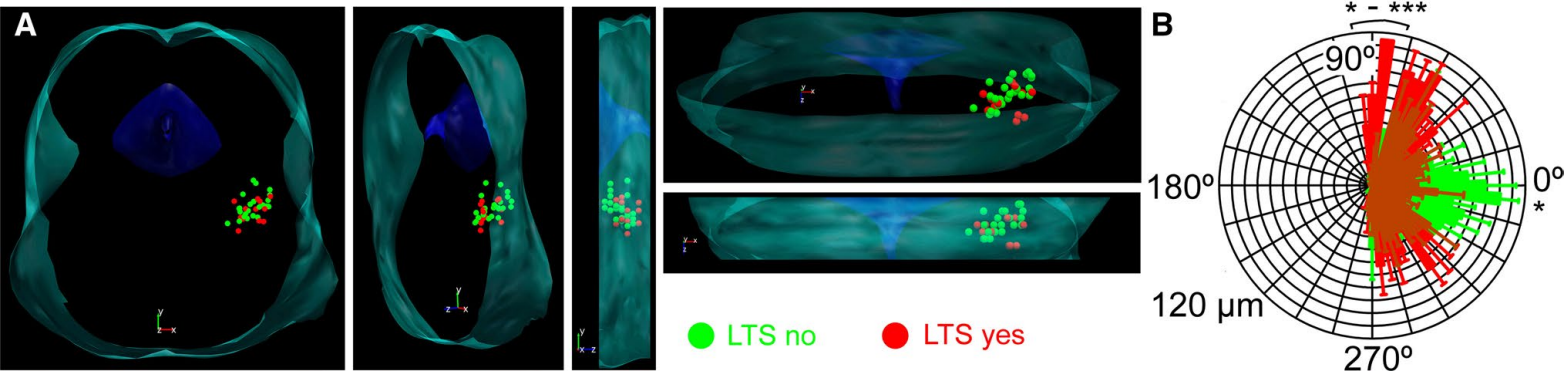
Morphological characteristics of neurons possessing or lacking low threshold spikes. **a** Distribution map of existence or absence of LTS in the PPN viewed from different angles (green = no LTS was detected; red = LTS exists). Note that the neurons with LTS are mostly located in the rostral PPN. **b** Averages of polar histograms from neurons possessing (red) and lacking (green) low threshold spikes. Asterisks represent significant differences between datasets belonging to certain angles

Among those neurons, where the rostrocaudal location of somata was unambiguously determined (*n* = 33), type I cholinergic neurons comprised of 12.12% (*n* = 4) of the total population, 45.45% (*n* = 15) were type II, whereas 21.21–21.21% (*n* = 7 in both cases) of the neurons fell to the type III and IIIK categories. In the caudal part of the PPN (the pars compacta; *n* = 13), type II neurons dominated (69.2%; *n* = 9), whereas 7.69–7.69% (*n* = 1 − 1) were type I and III. 15.38% of the neurons (*n* = 2) in the region were type ‘IIIK’. In the rostrally located pars dissipata, the proportions were different: 15% (*n* = 3 from 20) of the neurons were type I, 30–30% (*n* = 6 in both cases) were type II and III, whereas 25% (*n* = 5) fell to the category of type IIIK (Fig. 3e, f).

In the pars compacta, neurons with LTSs or rebound firing are underrepresented. In this region, 15.4% (*n* = 2 from 13) of all cholinergic neurons displayed this phenomenon, whereas in the pars dissipata, this proportion was 47.3% (*n* = 9 from 20; Fig. 3a). Interestingly, neurons possessing or lacking LTS had significant differences in the dendritic orientation. Neurons with LTS had bipolar dendritic tree, whereas the ones lacking LTS were rather multipolar (Fig. 3b).

According to further electrophysiological parameters not used for sorting PPN neurons, no significant differences were found in the input resistance and adaptation index of the subgroups (Suppl. Fig. 3).

**Fig. 4:**
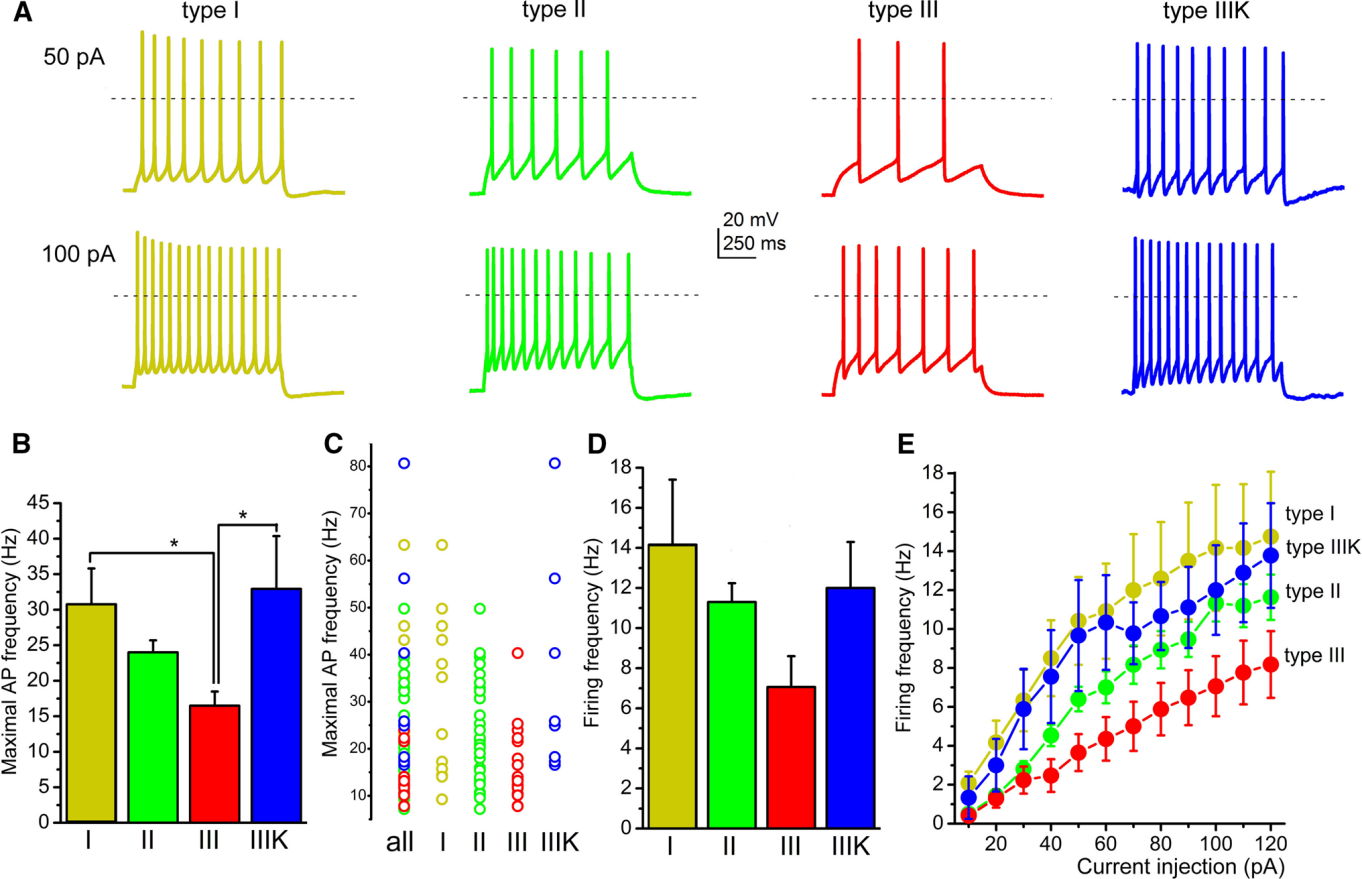
Type III neurons fire slower than other subgroups. **a** Voltage traces recorded with injection of 50 (upper row) or 100 pA square current injection (lower row), using the same color code as in Fig. 2. Dashed lines indicate 0 mV. **b** Statistical comparison of maximal detected action potential firing frequencies (typically calculated from the first two action potentials of a train elicited with 120 pA current injection). **c** Statistical analysis of firing frequencies for 1-s-long action potential trains elicited with 100 pA current injection. **d** Comparison of average firing frequencies obtained with square pulse injections with different magnitudes. Note that firing frequencies of type III neurons from 40 to 120 pA current injections are significantly different from firing frequencies of all other groups. **e** Individual data for maximal action potential firing frequencies with the same color code as used before

When maximal action potential firing frequency (calculated from the shortest interspike interval of a cell) or average firing frequency (calculated from the number of spikes in a 1-s-long trace) were assessed (from 91 neurons), type III neurons were the slowest firing, with significantly less maximal firing frequency. The maximal frequency of type III neurons was 16.49 ± 1.99 Hz, whereas it was 30.76 ± 5.04, 23.99 ± 1.69 and 32.94 ± 7.44 Hz for type I, II and IIIK, respectively (I–III, *p* = 0.031; III–IIIK = 0.02; Fig. 4a–c). Similar tendencies were seen with average firing frequencies. The average frequency of type III neurons during 100 pA current injection was 7.06 ± 1.54 Hz, which was numerically lower than the 14.16 ± 3.24, 11.3 ± 0.94 and 12 ± 2.3 Hz frequencies of type I, II and IIIK groups, respectively (Fig. 4d, e).

It is known that a small subpopulation of cholinergic neurons co-release glutamate and acetylcholine [25]. To check whether these neurons belong to a single functional group, 67 marker-positive neurons were patched from Vglut2-tdTomato mice. Post hoc choline acetyltransferase labelling revealed ChAT positivity of 3 neurons (4.47%; Suppl. Fig 4A). One of these neurons belonged to type I, whereas two of them were type II (Suppl. Fig 4B). Although the number of the cases is too low to know the proportions of functional cell types is this special population, one can still conclude that glutamatergic-cholinergic neurons do not belong to a single functional cell type.

### Early- and late-firing neurons of the PPN

**Fig. 5:**
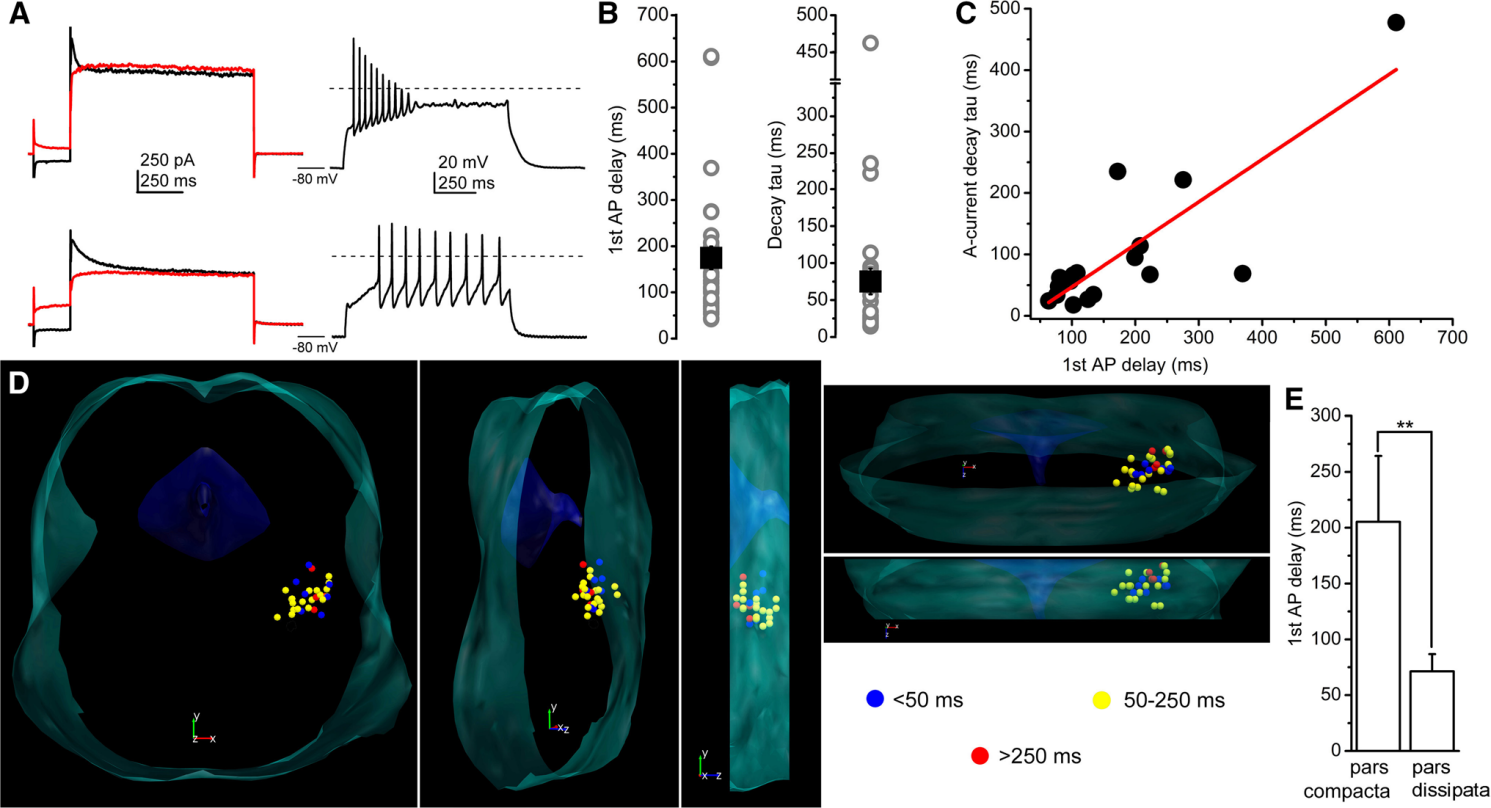
Neurons possessing transient outward current can be grouped as early- and late-firing neurons. **a** Current-and voltage traces recorded from an early-(upper row) and a late-firing neuron (lower row). Left: current traces elicited with depolarizing square voltage steps, preceded by hyperpolarizing (black) and depolarizing prepulses (red). Right: voltage traces recorded with 100 pA depolarizing square current injections from − 80 mV resting membrane potentials. **b** Statistical summary of the spike latency (recorded at − 80 mV resting membrane potential) and the decay tau of the declining phase of the transient outward currents (gray circles: individual data; black squares: average ± SEM). **c** Decay tau of the transient outward currents plotted against the spike latency at − 80 mV resting membrane potential (individual data: black squares; red line: linear fit). **d** Distribution map of spike latencies at − 80 mV resting membrane potentials (blue: latency below 50 ms—considered as neurons with no transient outward currents, yellow: latency between 50 and 250 ms—considered as early-firing neurons; red: longer latency than 250 ms—considered as late-firing neurons). Note that late-firing neurons are located caudally. **e** Statistical comparison of spike latencies from the pars compacta (caudal region) and pars dissipata (rostral region; average ± SEM)

We next focused on investigating neurons with greater firing latency than 50 ms from a hyperpolarized resting membrane potential. First, we tried to confirm that the presence of transient outward currents is the background of the spike latency. To assess this, action potential trains were elicited with 100 pA square current injections from − 80 mV. The spike latency evoked from − 80 mV with 100 pA current injection was 174.57 ± 24.7 ms (ranging from 57.3 to 718.9 ms) in this population. On the same neurons, after treatment with TTX, depolarization-activated outward currents were recorded with hyperpolarizing and depolarizing prepulses. Depolarizing prepulses abolished the transient currents, otherwise present with hyperpolarizing prepulses. The declining phase of the transient outward current was fitted with a single exponential function, with a decay tau of 75 ± 17.5 ms (from 14.3 to 477.4 ms; Fig. 5a, b). When the decay tau of the transient outward current was plotted against the spike latency recorded from the same neuron, a strong linear correlation was observed with a slope of 0.69 and a *R*-square value of 0.673 (*n* = 18; Fig. 5c).

When spatial distribution of the spike latency was assessed, it was found that neurons with longer latencies (above 250 ms) are located caudally, whereas rostrally located neurons had a shorter latency. The average latency of the neurons in the pars compacta was significantly longer than in the pars dissipata (205.3 ± 59 ms vs. 71.4 ± 15.5 ms; *p* = 0.0033; Fig. 5d, e).

Summarizing our findings with distributions of A-current kinetics and hyperpolarization-induced spike latencies, we suggest that neurons possessing A-current can be sorted into two groups:‘early-firing’ neurons with a latency shorter than 250 ms and ‘late-firing’ neurons with a latency exceeding 250 ms (as the basal forebrain cholinergic neurons; [40]). The latter group formed a minority of the cholinergic neurons with A-current (19%, *n* = 4 from 21), and was located caudally.

### High threshold membrane potential oscillations

We next investigated another functional characteristic of PPN cholinergic neurons, the HTOs. These oscillations are determined by different sets of voltage-gated calcium and potassium channels and characteristics of cholinergic, but not GABAergic and non-cholinergic neurons [24, 25, 35]. We confirmed the previous findings that cholinergic neurons display this phenomenon. According to our data, HTO activity did not differ among the classical functional groups. However, when the power maxima and the frequencies belonging to HTO activity were compared with the location of the neuronal somata, we found that high frequency–low power oscillations are characteristic for the pars compacta and low frequency–high power oscillations are found in the pars dissipata. In the pars compacta, 6.59 ± 3.8 mV^2^/Hz power was coupled with 23.07 ± 4.9 Hz frequency, whereas 18.85 ± 5.08 mV^2^/Hz power was measured with 12.08 ± 2.01 Hz frequency in the pars dissipata (*n* = 10).

**Fig. 6:**
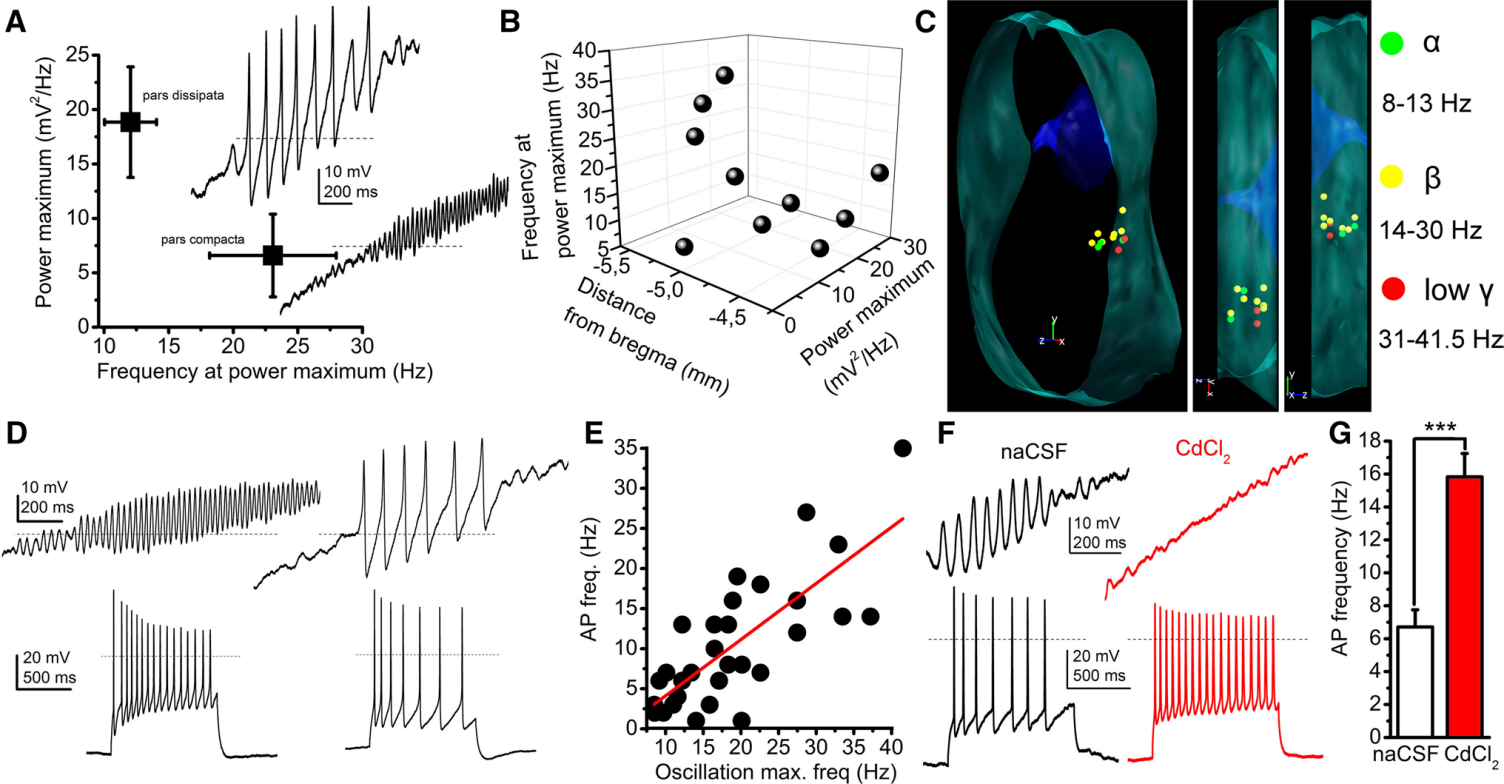
High threshold membrane potential oscillations (HTOs) of PPN neurons determine action potential firing frequencies. **a** Average frequencies of the power maximum plotted against the oscillation frequencies recorded at the power maximum of neurons in the pars compacta (below) and pars dissipata (above). Original voltage traces (right) were elicited by depolarizing ramp current injections in the presence of TTX. Dashed lines indicate 0 mV. **b** Spatial distribution of HTO frequencies at peak power maximum and power maxima. **c** Distribution map of oscillatory frequencies at power maximum (green: theta/alpha; yellow: beta, red: low gamma frequency range). **d** Relationships of oscillatory frequencies and action potential firing frequencies. Upper row: HTOs elicited with depolarizing ramp injection. Lower row: action potential trains recorded from the same neurons as upper traces, with 120 pA depolarizing square pulses. **e** Action potential firing frequencies plotted against oscillatory frequencies at power maximum (black circles: individual data, red line: linear fit). **f** Changes of firing frequency with application of 50 µM CdCl_2_. Upper traces: HTOs under control conditions (black) and in the presence of CdCl_2_ (red), recorded from the same neuron. Lower traces: action potential trains recorded under control conditions (black) and in the presence of CdCl_2_ (red). **g** Statistical comparison of action potential firing frequencies without and with CdCl_2_ (average ± SEM)

Cholinergic neurons possessing HTOs matching the beta frequency range were found all along the rostrocaudal axis of the PPN, whereas neurons with membrane potential oscillations in the theta/alpha frequency range were found rostrally, cholinergic cells possessing HTOs in the low gamma frequency range were found in the caudal region of the nucleus (Fig. 6a–c).

When the significance of these oscillations on action potential firing frequency was investigated, a strong linear correlation was found between action potential firing frequency evoked by 120 pA depolarizing square current injection and the oscillatory frequency (slope: 0.702, *R*-square: 0.573; *n* = 30; Fig. 6d, e). As a further confirmation of this finding, when oscillations were blocked by 50 µM CdCl_2_, the action potential firing frequency significantly increased (from 6.71 ± 1.04 Hz to 15.83 ± 1.42 Hz;* p* = 0.0006; *n* = 7; Fig. 6f, g; Suppl. Fig 5).

**Fig. 7:**
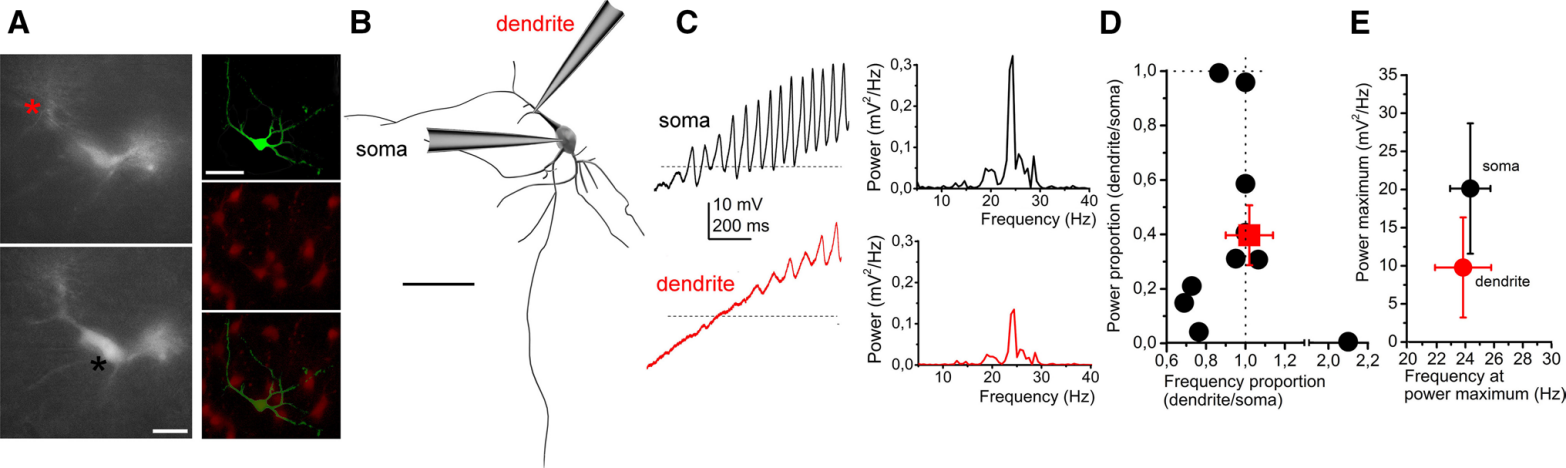
High threshold oscillations (HTOs) are generated by the somata. **a** Morphological situations of soma-dendrite paired recordings of a PPN cholinergic neuron. Left: wide field image of the in situ localization of the recording electrodes in different focal planes. Dendritic (above, red) and somatic patch electrodes (below, black) are indicated with asterisks. Right: Post hoc visualization of the recorded neuron (above: biocytin labeling, middle: tdTomato expression; below: merged image; scale bar: 50 µm). **b** Post hoc reconstruction of the neuron of panel A, with the places of recording (scale bar: 50 µm). **c** HTOs recorded from the soma (above, black) and the dendrite (below, red) of the reconstructed neuron on panel B. Individual power spectra belonging to the traces are below the traces. **d** Statistical analysis of power and frequency proportions of dendritic and somatic oscillations (individual values obtained from dendrites are divided with individual values recorded from somata of the same neuron; black circles: individual data; red square: average ± SEM). Dashed lines indicate 1. **e** Averages of somatic (black) and dendritic (red) values

Furthermore, the origin of these membrane potential oscillations was also sought and simultaneous soma-dendrite patch-clamp recordings were performed. The average distance of the dendritic recording site from the origin of the dendrite was 21.44 ± 4.96 µm. We found that dendritic oscillations had a lower power but almost identical frequency (20.13 ± 8.55 mV^2^/Hz power and 24.34 ± 1.41 Hz for the soma and 9.77 ± 6.57 mV^2^/Hz for the dendrite). Although the difference in power was not significant (due to high variance), it was seen that the dendritic oscillatory power was 39.7 ± 12% of the somatic, whereas the frequency was almost the same (102 ± 11%; Fig. 7a–e). This power decay towards dendritic segments was similar to the decremental propagation of electrical impulses from the soma to the dendrites (when a square current pulse was injected to the soma and voltage changes were recorded at both sites, 42.4 ± 4% of the somatic voltage change appeared on the proximal dendrites; *n* = 10).

Taken together, we found that neurons of the pars compacta and pars dissipata are oscillating on different frequencies. These oscillations have greater amplitude on the soma and seem to be reduced on the proximal dendrites. Furthermore, oscillatory activity correlates with the firing rate of the neurons.

## Discussion

In the present paper, we provided evidence that ChAT-tdTomato model is suitable for investigating cholinergic neurons and previous data on functional neuronal groups can be confirmed using this animal model. It was also confirmed that HTOs exist on cholinergic neurons and they are related to action potential firing frequency. Besides obtaining confirmatory data of previous studies, it was demonstrated that ‘canonical’ neuronal subgroups have different proportions in the rostral and caudal parts of the PPN. LTSs rarely lead to action potential firing of cholinergic neurons and are properties of neurons in the rostral PPN. Neurons possessing A-current (type II and III) can be subdivided to ‘early-’ and ‘late-firing’ neurons, where the latter ones are located caudally. HTOs have high frequency and low power in the caudal, and low frequency, high power in the rostral PPN. Furthermore, HTOs have greater amplitude on the somata than in the proximal dendrites.

### Neurochemical identification of PPN cholinergic neurons

The majority of the functional studies on the PPN characterize neurons in terms of their neurochemical properties using various methods. In most in vivo and slice electrophysiological studies (characteristically, but not exclusively by studies before the emergence of transgenic animal techniques), post hoc neuronal identification and immunolabeling is used. Both in vivo [3, 41] and slice electrophysiology studies [19] use NADPH diaphorase or bNOS labeling [39]. The almost full overlap between ChAT and bNOS labeling is well demonstrated [21, 42]. Probably, the most common way of identifying cholinergic neurons of the PPN is the ChAT immunohistochemistry: several slice electrophysiology studies [12, 14–18, 22, 23, 43, 44], as well as in vivo experimental approaches [6, 27] employed it. As alternative methods, vesicular acetylcholine transporter (VAChT) immunolabeling [26] or single cell RT-PCR for detecting ChAT was also used [20].

After transgenic techniques became available for rats and mice, several in vivo approaches identified cholinergic neurons using cre-expressing animals under ChAT promoter. After being injected with cre-dependent viruses, these animals expressed channelrhodopsin 2 tagged with YFP [28, 29, 45, 46] or mCherry, as well as hM3 tagged with mCherry [47], demonstrating robust development in selective activation of neuronal subgroups of PPN and assessment of their functions.

Until now, although efforts for recordings on genetically identified cholinergic neurons were done to demonstrate the existence of M-current [25], spike frequency adaptation [48] or neuromodulatory actions [49, 50], systematic analysis of membrane properties on genetically identified PPN cholinergic neurons did not exist. In line with the literature data [50], we demonstrated that the ChAT-tdTomato mouse model can be effectively used for functional studies. A negligible proportion of tdTomato-positive neurons was proved to be ChAT-negative with immunohistochemistry. However, there is a population of neurons forming 11% of all labeled somata which displayed ChAT positivity by immunohistochemistry and did not express tdTomato. This finding raises the possibility that a distinct subgroup of cholinergic neurons is excluded from analysis when genetically identified cholinergic neurons are used. The single study using both labeling for identification of cholinergic neurons does not support this concern: neurons identified with tdTomato expression or immunohistochemistry possessed or lacked M-current in an identical way [25]. In conclusion, the present study demonstrates that using mouse models where cholinergic neurons are genetically identified is a reliable tool for assessing neurochemical identity of neurons.

### ‘Classical’ functional subgroups of PPN cholinergic neurons

PPN neurons are traditionally classified to three groups according to their membrane properties. Type I neurons have low threshold spikes (due to calcium conductances), which enables them to burst firing. Type II neurons have A-current, whereas type III neurons have both properties [18, 19, 37, 38] or none of them [22]. The latter group is sometimes referred as type IV [39]. This latter type is marked as IIIK by us, where ‘K’ indicates the acronym of the authors who first described this neuronal type [22]. In the following text, we use the terms according to our classification, “translating” classifications of the actually cited literature to ours; although it was not always originally referred like it.

Type II neurons are referred by most authors as the most common type of the PPN [18, 37, 39], as the proportion of them ranged between 52.2 and 75%. In contrast, Kang and Kitai [22] found that only one-third of the neurons can be classified to this group. The cholinergic or non-cholinergic nature of the neurons was also examined by these studies and found that all type II neurons are cholinergic [22], non-cholinergic neurons fell into group I, 82% of them were type II and 18% was type III [18]. In another study, 3% proved to be type I, 75% was type II and 22% was type III [37]. It is worth noting that all studies were performed on genetically non-manipulated rats.

Being roughly in consensus with the literature, our study on transgenic mice showed that only a minority (12%) of the neurons was type I and most neurons fell to the type II category (48%). In addition, it was found that type II neurons have an even higher proportion in the caudal PPN, whereas only one-third of the neurons belong to this group rostrally. This finding is in contrast with previous data [18] reporting that type II neurons are rather rostrally located. The reason of the discrepancy is possibly the fact that, first, non-cholinergic neurons were also considered by that study; second, interspecies differences might exist in this feature. A clear rostrocaudal gradient of low threshold spikes was also observed by us: LTS seems to be rather characteristic for the rostral PPN. Of note, possessing LTS does not mean that these neurons are bursting: less than 10% of the neurons fired a single action potential on the peak of the LTS and two or more action potentials were never observed. In contrast, a greater subpopulation of glutamatergic PPN neurons were able to fire bursts (unpublished data). Furthermore, the Vglut2- and ChAT-positive small subpopulation of PPN neurons does not seem to be homogenous or identical with any functional subgroups.

Neurons belonging to different types were found to be morphologically distinct in terms of soma area and dendritic number [18, 22]. We found that neurons with LTS had a bipolar dendritic tree, whereas the ones lacking LTS were multipolar. Out of this, no meaningful correlations were revealed by the present study between types or other functional parameters and morphometric data. According to functional differences out of low threshold spikes and A-current, the only finding was that thalamic-projecting type II neurons tended to be inhibited by carbachol or exerted biphasic response, whereas type III neurons were stimulated by it [39]. The resting membrane potential of different neuronal types was statistically not different [35]. We also tested several functional data of neurons falling to different types and found that the input resistance, spike frequency adaptation or HTOs did not show any significant differences. Notably, the only difference was that type III neurons fired with a significantly lower maximal frequency to depolarizing stimuli.

Based on our results, one can conclude that the ‘canonical’ sorting of PPN neurons can still be used for describing membrane properties of these neurons. Rostrocaudal differences of LTS and low firing rate of type III neurons together may suggest a functional separation of cholinergic neurons along the rostrocaudal axis (see below).

### Early- and late-firing PPN cholinergic neurons

An important feature of type II and III neurons is the presence of A-current. It is a transient, voltage-gated potassium current which is thought to determine spike latency after application of a depolarizing stimulus [33, 34, 40]. A-current is measurable after application of a depolarizing stimulus preceded by hyperpolarization, which removes inactivation of the current [33, 51]. Thus, when neurons with A-current are hyperpolarized, spike latency is expected to be longer. The decay time constant of the A-current can vary between 50 and 500 ms. Currents with slower kinetics are considered as “slow” A-current; which is sometimes referred as D-current [40, 52].

Spike latency is supposed to correlate with A-current decay time constant, except if other conductances are activated in concert with it and modify its action on changes of the resting membrane potential. On the basal forebrain cholinergic neurons, early-firing and late-firing neurons were distinguished but no correlation between this grouping and A-current decay tau was shown [40, 53]. Similar to this classification, we propose that early- and late-firing populations of PPN neurons possessing A-current can be distinguished. In contrast with the basal forebrain, a clear correlation can be seen between A-current kinetics and spike latency in the PPN. Representing another rostrocaudal gradient of functional properties, the smaller subgroup of late-firing neurons is caudally located.

Besides spike latency of a neuronal population under physiological conditions, one might speculate about the pathophysiological vulnerability of neurons normally possessing A-current with greater amplitude. It was found that models of inflammatory conditions reduce A-current amplitude of cardiomyocytes [54]. It might be an inviting hypothesis that neuroinflammatory conditions affect firing pattern of PPN cholinergic neurons by reducing A-current and thus spike latency, contributing to altered activity cycles.

### High threshold membrane potential oscillations

Intrinsic membrane potential oscillations are present in several brain areas, best known in the entorhinal cortex and neocortex [55, 56], amygdala [57], thalamus [58] and parabrachial area [59]. Intrinsic oscillations are present on PPN neurons, as well [23–25, 35]. Certain oscillatory activities are TTX-sensitive [35], whereas others are TTX-resistant [24, 25]. TTX-insensitive HTOs can be recorded at highly depolarized membrane potentials (− 30 to + 15 mV) and mediated by P/Q and N-type calcium channels [24, 60], and dendrotoxin-sensitive [24] and M-type [25] potassium channels.

Frequencies of the oscillations vary between 4–16 Hz [23] and 4–80 Hz [24], from theta to beta-gamma range. High amplitude–low frequency oscillations are found to be rostral, whereas the opposite (i.e., low amplitude–high frequency) is the property of the caudal PPN [23].

In the present study, it was demonstrated that cholinergic neurons—independently from the classical functional subtypes or morphological features—possess membrane potential HTOs. Confirming the original findings of Takakusaki et al. [23], HTOs with low frequency and high amplitude are found on rostrally located genetically identified cholinergic neurons, whereas caudally located neurons possessed high frequency–low amplitude activity.

The main place of generation for HTOs was suggested to be the distal region of dendrites [61, 62]. To assess this question, we performed parallel recordings from the soma and a dendrite. Because of technical limitations; we did not check distal but the proximal dendrites approximately 20 µm away from their origin. We found that the somatic oscillatory activity appears in the dendrites with reduced amplitude but with the same frequency and timing. Therefore, one can hypothesize that the place of HTO generation might be the soma and electrical changes passively propagate towards the proximal dendrite. However, based on the present data, one cannot exclude that distal dendrites have own oscillatory activity.

The physiological function of these oscillations is also an important question. It is thought that membrane potential oscillations in the PPN are responsible for gamma oscillations of the nucleus and finally determine gamma oscillatory activity in the cortex [24]. This hypothesis might be supported by findings in the cortex, where interneuronal intrinsic membrane properties can determine gamma activity [63]. However, studies on Parkinsonian patients underwent deep brain stimulation do not clearly support overwhelming gamma oscillatory activity of the PPN. Only a single study supports existence of gamma activity [64], whereas alpha and beta activities were demonstrated by several others [11, 65–70]. Interestingly, there is a contrast between the rostrocaudal distribution of oscillatory frequencies measured from a PPN subregion in humans and recorded from individual rodent neurons. In human PPN activity, the lower frequencies were recorded caudally, opposite to our studies and literature data from individual neurons from rodents. Contradictions between patch-clamp studies on rodents and recordings from human PPN might indicate that either there is a more complex relationship between intrinsic membrane oscillations and local field potentials or global brain states than previously hypothesized, or important interspecies differences are present.

This study also demonstrated that HTO frequency is directly proportional to the average firing rate of the same neuron and inhibition of HTOs significantly increases firing rate. Mechanistically thinking, it is hard to imagine the influence of a phenomenon appearing from − 30 mV membrane potential on an ongoing action potential. The key link between the firing rate and HTOs is possibly the ion channel set of the membrane determining both phenomena; from which action potentials can be recorded with an arrangement closer to physiological and an experimental setting being more artificial (presence of TTX, depolarizing ramp protocol). Furthermore, determination of the firing rate probably also argues for the somatic origin of HTOs; as action potentials are most effectively affected by a set of ion channels close to the axon hillock.

Taken together, we confirmed the existence and rostrocaudal differences of HTOs. We also found that HTOs are rather generated in the soma and not on the dendrites; and this phenomenon determines action potential firing rate of the neurons.

### Topographical differences of the functional properties

The PPN has a well defined topographical organization in terms of neuronal projections. The caudal PPN gives rise to fibers targeting ventral tegmental area and dorsomedial striatum, whereas the rostral PPN innervates the pars compacta of the substantia nigra and the dorsolateral striatum [2, 28, 29]. These findings are in accordance with the reported functional differences between the rostral and caudal portions of the PPN. Acetylcholine-induced REM sleep was elicited with a shorter delay and higher percentage when injected to the rostral PPN than to the caudal [30]. In humans, the dorsal PPN had an increased activity while subjects had fast imaginary walking task, and the ventral PPN was activated in parallel with imagining objects moving with a high velocity [31]. Pathophysiological studies also revealed rostrocaudal differences: deep brain stimulation of the rostral PPN in a Parkinson’s disease (PD) model worsened gait and led to freezing, whereas caudal PPN stimulation led to mild gait improvement [6]. In another PD model, the number of cholinergic neurons was significantly decreased and the galanin-containing population was significantly increased in the rostral PPN but not (or in a much less extent) in the caudal part of the nucleus [17].

We found several membrane properties showing rostrocaudal differences. Possessing LTSs is a property of the rostrally located cholinergic neurons, whereas late-firing ones with slower A-current are characteristic for a subgroup of caudal PPN cholinergic neurons. Important rostrocaudal differences it HTOs, thus finally firing rates were also demonstrated. These properties might serve as backgrounds of previous in vivo and in vitro findings demonstrated above.

### Concluding remarks

In conclusion, ‘classical’ electrophysiological grouping of PPN neurons is physiologically meaningful and still capable of sorting them. Similarly, ChAT-tdTomato mice are good tools for identification of cholinergic neurons and results from them are comparable with previous results despite of interspecies differences and different experimental arrangements.

Besides the traditionally used electrophysiological neuronal subgroups, other electrophysiological markers might also be suitable for defining functional groups of PPN cholinergic neurons. First, one can distinguish neurons with high frequency–low amplitude and low frequency–high amplitude HTOs. Second, according to the decay time of A-current and the duration of spike latency, one can distinguish neurons lacking A-current, or being ‘early-firing’ or ‘late-firing’.

Marked rostrocaudal differences of several membrane properties were also reported. LTSs are predominantly found rostrally, whereas ‘late-firing’ neurons are in a caudal location. High frequency–low amplitude HTOs (in the beta-gamma range) appear caudally, whereas low frequency–high amplitude HTOs (in the alpha range) are rostral. These in vitro functional differences demonstrate the cellular background of in vivo experimental or clinical findings with rostrocaudal distribution of various parameters (see above).

Finally, we investigated the functional role and place of generation of HTOs. We concluded that HTO frequency has a strong correlation with spike frequency. This direct measurements from the dendrites support our suggestion that these phenomena are rather generated on the soma than on the dendritic tree.

We believe that these in vitro findings provide help for design and analysis of in vivo experiments using transgenic models, as well as for understanding different outcomes of DBS in various locations along the PPN.

### Electronic supplementary material

Below is the link to the electronic supplementary material.

**Supplementary Fig.** **1. Assessment of different electrophysiological parameters of PPN cholinergic neurons. A.** Recording of the input resistance. Above: current protocol (1-s-long hyperpolarizing square pulse with -30 pA amplitude). Below: voltage trace obtained with the current protocol above. Red lines indicate the segments of the trace where data points were averaged for calculation of voltage differences. **B.** Calculation of the adaptation index (AI). F_last_ is the average frequency of the last two action potentials and F_initial_ is the average frequency of the first three action potentials (indicated with arrows and red dashed lines). **C.** Recording of the transient outward potassium current. Above: voltage protocol used for A-current recording. Black: protocol with hyperpolarizing prepulse. Red: protocol with depolarizing prepulse. Below: current traces obtained with the protocol above. Black: current recorded with a hyperpolarizing prepulse. Red: current recorded with a depolarizing prepulse. Right: Single exponential fit of the declining phase of the recorded current. **D-F.** Assessment of the HTOs. **D.** Depolarizing ramp current protocol (above) and voltage trace recorded in naCSF. Note that the HTOs are partially covered with action potentials. **E.** Voltage trace recorded in the presence of TTX. **F.** Power spectrum of the trace shown on panel E. Red dot and red dashed lines indicate the parameters considered for further analysis. (JPEG 799 kb)

**Supplementary Fig.** **2. Correlations of morphometric, topographic and functional data. A.** Overview of the gross anatomy of the PPN. Arrows and numbers indicate the distance from the bregma (based on Paxinos atlas [36]). **B-C.** No correlation was found between the dendritic length or the soma diameter and the rostrocaudal location of the neuronal somata. **D.** The soma diameter is inversely proportional with the input resistance (R-square: 0.307). **E.** The number of dendritic nodes and ends are directly proportional with the dendritic length (R-square: 0.63 and 0.71, respectively) (JPEG 858 kb)

**Supplementary Fig.** **3. Statistical analysis of the adaptation index and input resistance of PPN cholinergic neurons belonging to different functional subgroups.** For color codes please see Fig. 2. (JPEG 378 kb)

**Supplementary Fig.** **4. Examples for functional properties of Vglut2- and ChAT-positive neuronal subpopulation. A-D.** Assessment of the neurochemical identity of glutamatergic-cholinergic neurons. **A.** Biocytin labelling. **B.** Vglut2-dependent tdTomato expression. **C.** Post hoc ChAT labelling. **D.** Merged image. Scale bar = 50 µm. The arrows of panels B and C indicate the soma labelled with biocytin. **E-F.** Representative current clamp traces from a type I (**E,** yellow) and a type II (**F,** green) neuron recorded with 100 and 30 pA current injections (upper and lower traces, respectively) from -80 mV membrane potential. (JPEG 1186 kb)

**Supplementary Fig.** **5. Application of CdCl**_**2**_**slowly eliminates HTOs. A.** Voltage traces recorded with ramp current injection with TTX and with adding CdCl_2_. **B.** Power spectra of the traces on panel A, using the same color code. (JPEG 718 kb)
