# Supplementary figures and images for: Characterization of functional subgroups among genetically identified cholinergic neurons in the pedunculopontine nucleus
Source: Cell Mol Life Sci. 2019 Feb 8;76(14):2799–815. doi: 10.1007/s00018-019-03025-4 (PMC6588655; doi:10.1007/s00018-019-03025-4)

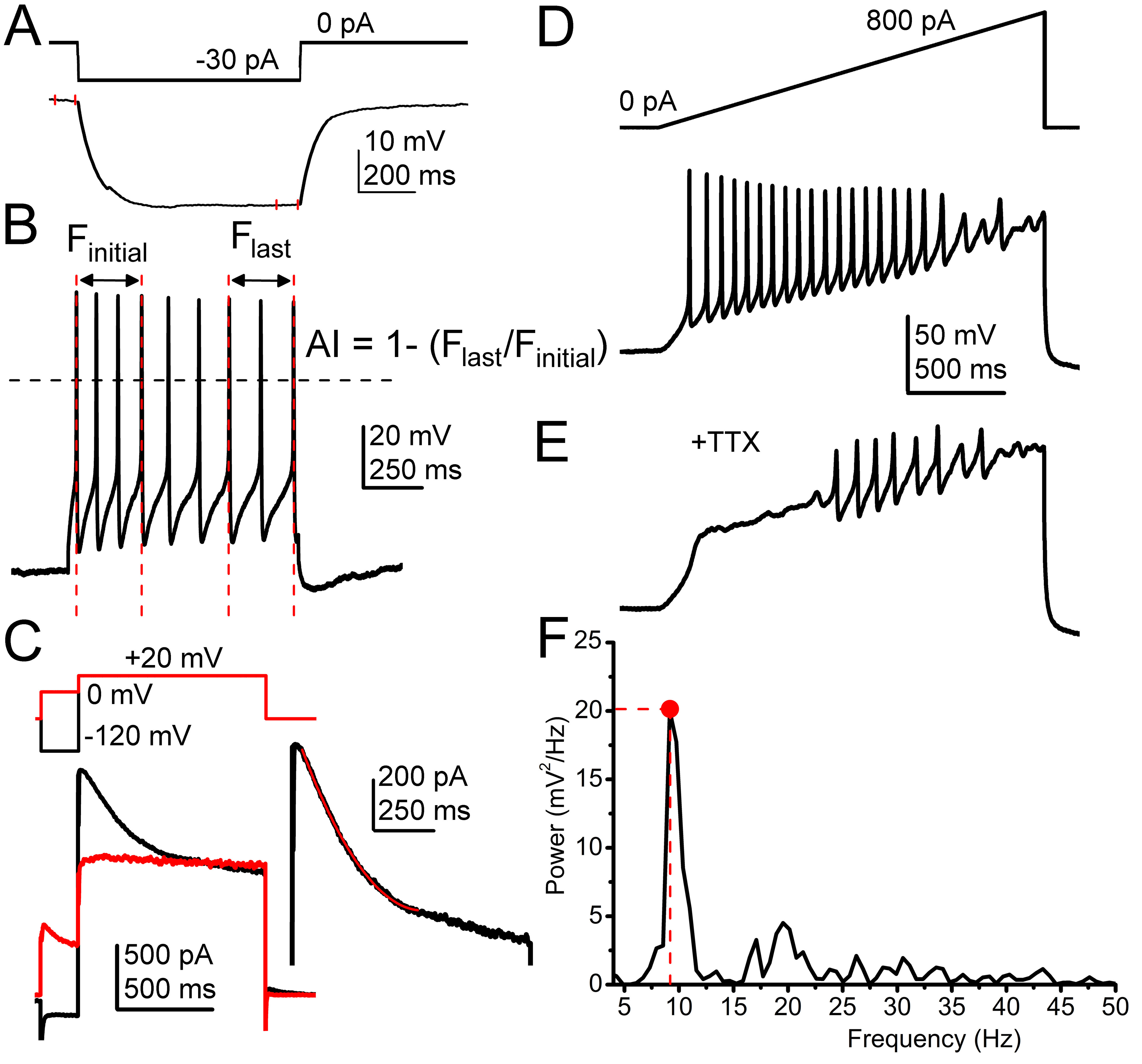

Supplement: Supplementary file 1 — Supplementary Fig. 1. Assessment of different electrophysiological parameters of PPN cholinergic neurons. A. Recording of the input resistance. Above: current protocol (1-s-long hyperpolarizing square pulse with -30 pA amplitude). Below: voltage trace obtained with the current protocol above. Red lines indicate the segments of the trace where data points were averaged for calculation of voltage differences. B. Calculation of the adaptation index (AI). Flast is the average frequency of the last two action potentials and Finitial is the average frequency of the first three action potentials (indicated with arrows and red dashed lines). C. Recording of the transient outward potassium current. Above: voltage protocol used for A-current recording. Black: protocol with hyperpolarizing prepulse. Red: protocol with depolarizing prepulse. Below: current traces obtained with the protocol above. Black: current recorded with a hyperpolarizing prepulse. Red: current recorded with a depolarizing prepulse. Right: Single exponential fit of the declining phase of the recorded current. D-F. Assessment of the HTOs. D. Depolarizing ramp current protocol (above) and voltage trace recorded in naCSF. Note that the HTOs are partially covered with action potentials. E. Voltage trace recorded in the presence of TTX. F. Power spectrum of the trace shown on panel E. Red dot and red dashed lines indicate the parameters considered for further analysis. (JPEG 799 kb) [file 18_2019_3025_MOESM1_ESM.jpg]

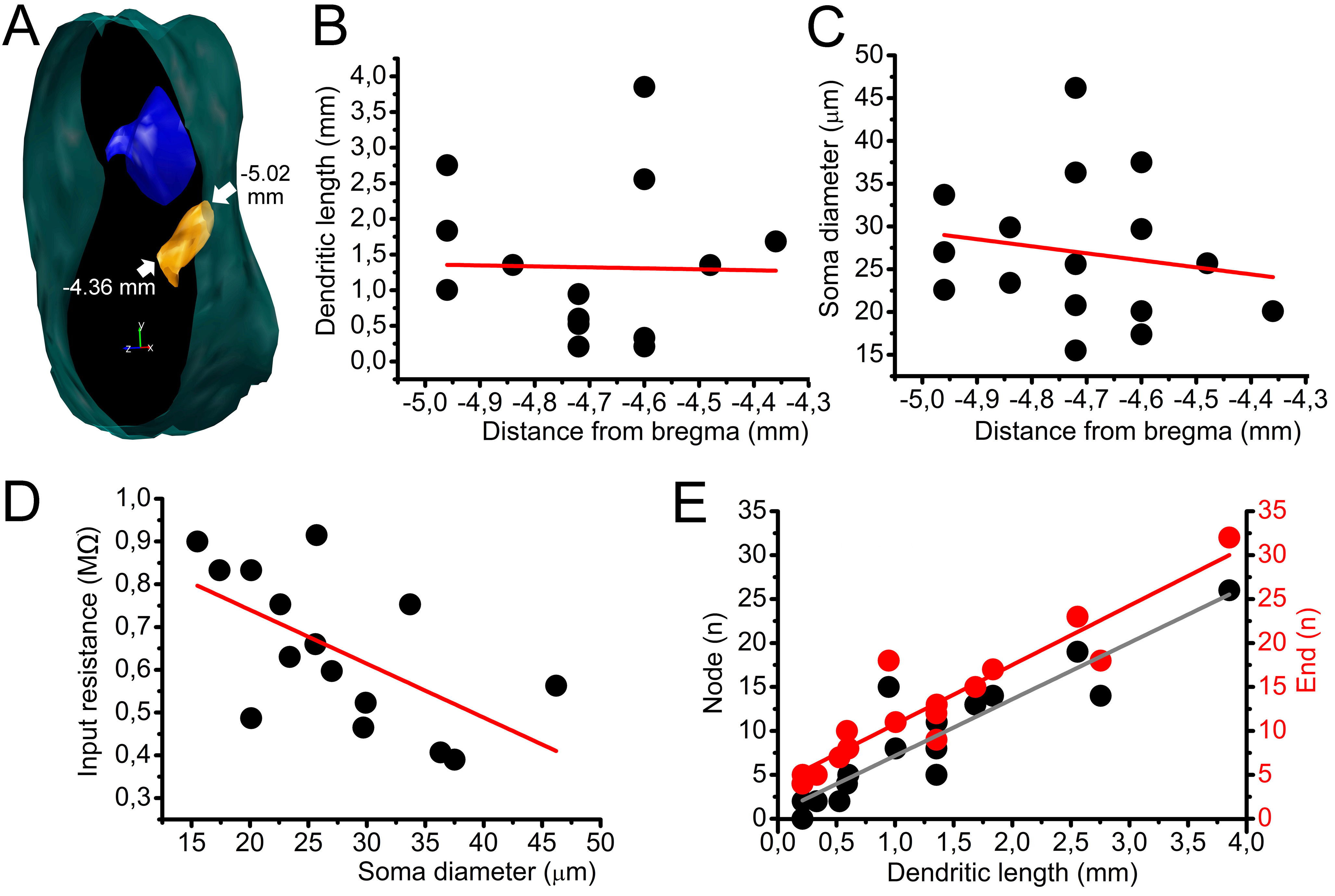

Supplement: Supplementary file 2 — Supplementary Fig. 2. Correlations of morphometric, topographic and functional data. A. Overview of the gross anatomy of the PPN. Arrows and numbers indicate the distance from the bregma (based on Paxinos atlas [36]). B-C. No correlation was found between the dendritic length or the soma diameter and the rostrocaudal location of the neuronal somata. D. The soma diameter is inversely proportional with the input resistance (R-square: 0.307). E. The number of dendritic nodes and ends are directly proportional with the dendritic length (R-square: 0.63 and 0.71, respectively) (JPEG 858 kb) [file 18_2019_3025_MOESM2_ESM.jpg]

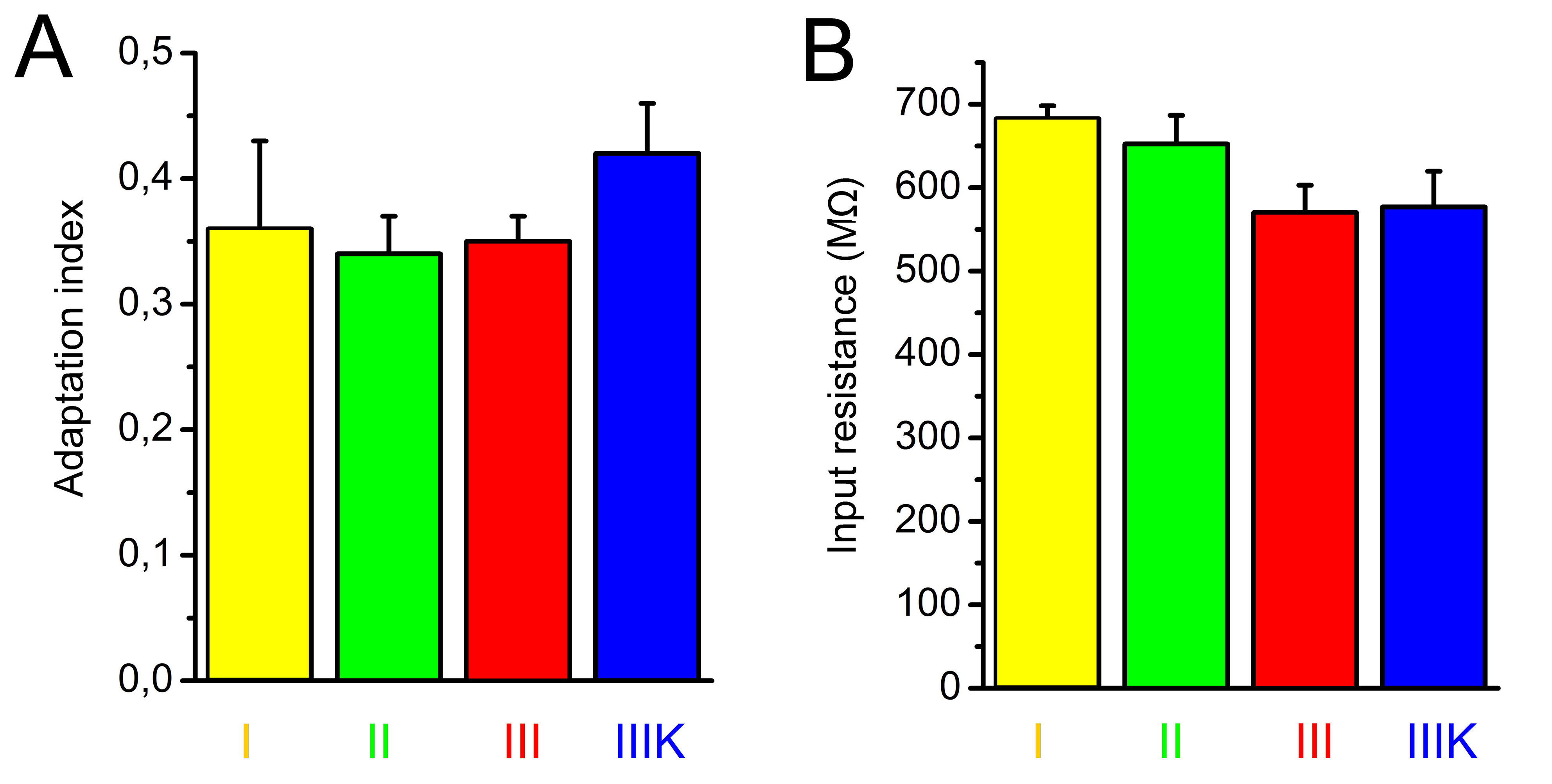

Supplement: Supplementary file 3 — Supplementary Fig. 3. Statistical analysis of the adaptation index and input resistance of PPN cholinergic neurons belonging to different functional subgroups. For color codes please see Fig. 2. (JPEG 378 kb) [file 18_2019_3025_MOESM3_ESM.jpg]

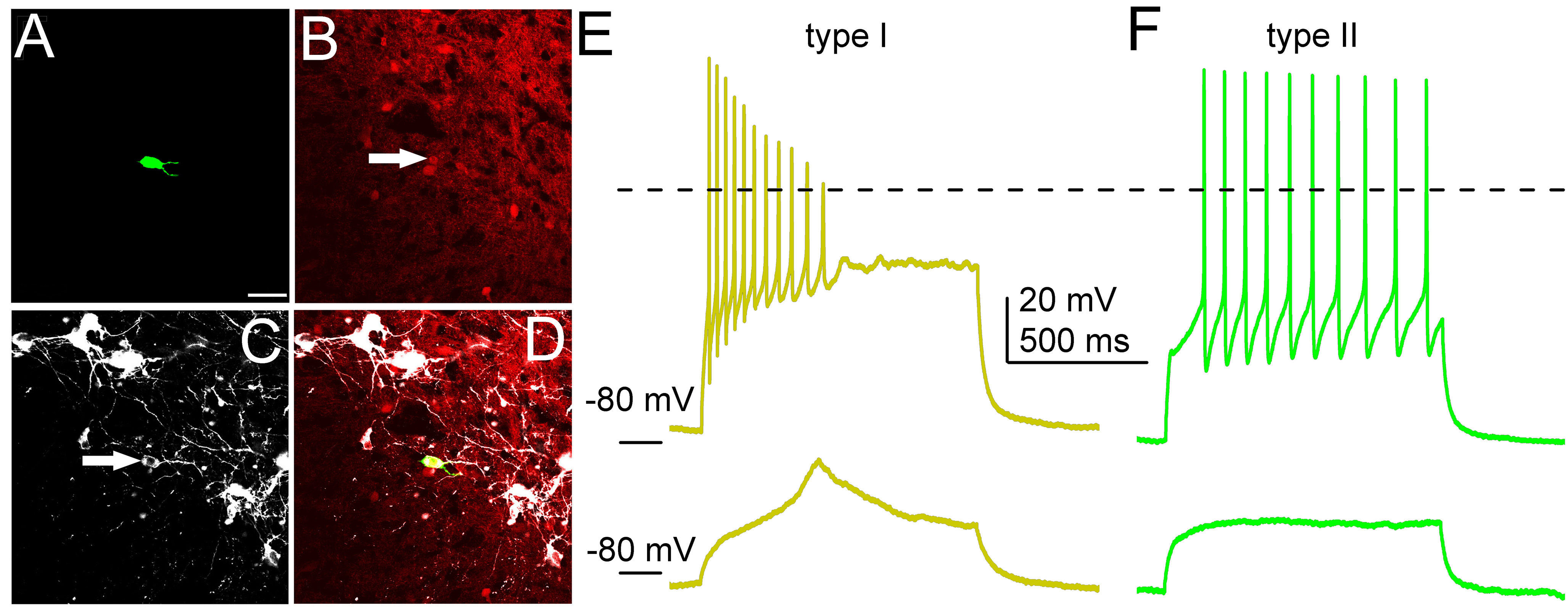

Supplement: Supplementary file 4 — Supplementary Fig. 4. Examples for functional properties of Vglut2- and ChAT-positive neuronal subpopulation. A-D. Assessment of the neurochemical identity of glutamatergic-cholinergic neurons. A. Biocytin labelling. B. Vglut2-dependent tdTomato expression. C. Post hoc ChAT labelling. D. Merged image. Scale bar = 50 µm. The arrows of panels B and C indicate the soma labelled with biocytin. E-F. Representative current clamp traces from a type I (E, yellow) and a type II (F, green) neuron recorded with 100 and 30 pA current injections (upper and lower traces, respectively) from -80 mV membrane potential. (JPEG 1186 kb) [file 18_2019_3025_MOESM4_ESM.jpg]

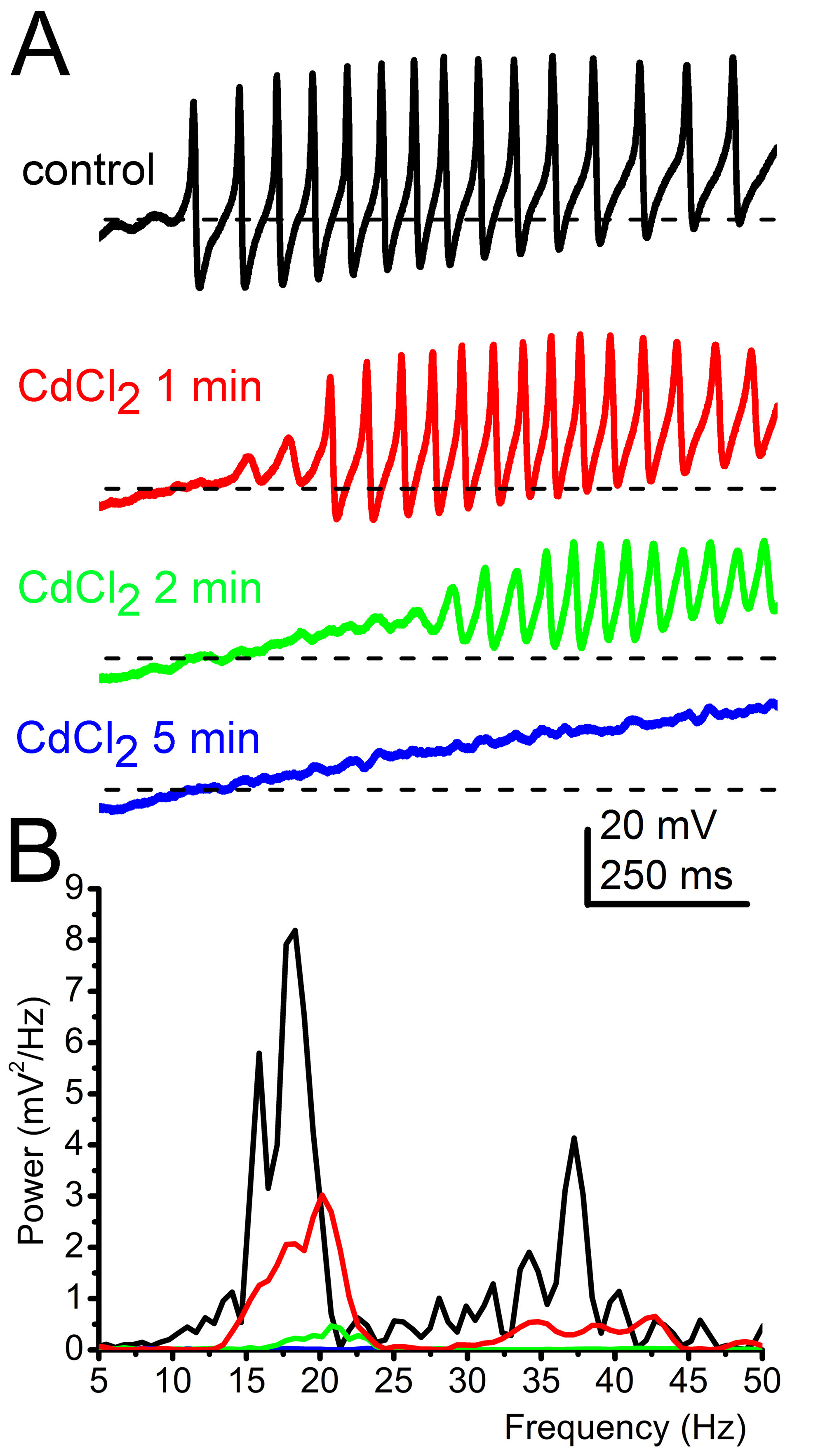

Supplement: Supplementary file 5 — Supplementary Fig. 5. Application of CdCl2slowly eliminates HTOs. A. Voltage traces recorded with ramp current injection with TTX and with adding CdCl2. B. Power spectra of the traces on panel A, using the same color code. (JPEG 718 kb) [file 18_2019_3025_MOESM5_ESM.jpg]
